# Academic performance of students in an accelerated medical pathway

**DOI:** 10.1080/10872981.2024.2345444

**Published:** 2024-04-28

**Authors:** Fei Chen, Katherine A. Jordan, Winston Li, Yee Lam, Luigi Pascarella, Catherine L. Coe

**Affiliations:** a Department of Anesthesiology, University of North Carolina, Chapel Hill, NC, USA; b Department of Pediatrics, University of North Carolina School of Medicine, Chapel Hill, NC, USA; c Department of Psychiatry, University of North Carolina School of Medicine, Chapel Hill, NC, USA; d Department of Family Medicine, University of North Carolina School of Medicine, Chapel Hill, NC, USA; e Department of Surgery, University of North Carolina School of Medicine, Chapel Hill, NC, USA

**Keywords:** Accelerated medical pathway, medical education, academic performance, residency readiness, assessment and evaluation

## Abstract

Accelerated medical school curricula, such as three-year programs, have gained attention in recent years but studies evaluating their impact are still scarce. This study examines the Fully Integrated Readiness for Service Training (FIRST) program, a three-year accelerated pathway, to assess its impact on students’ academic performance preparedness for residency. In this observational study, we compared the academic outcomes of FIRST program students to traditional four-year curriculum students from 2018 to 2023. We analyzed multiple metrics, including exam performance (United States Medical Licensing Examination Step scores, shelf exam scores, and pre-clinical course scores) and clinical performance scores during the application and individualization phases. Analysis of Variance was used to examine the effect of accelerated pathway program experience relative to traditional 4-year medical school curriculum on the learning outcomes. FIRST program students were on average 1.5 years younger upon graduation than their traditional peers. While FIRST program students scored slightly lower on Step 2 Clinical Knowledge (CK), they exhibited no significant differences in other exam scores or clinical performance relative to the traditional students. Notably, FIRST students achieved equivalent clinical performance ratings during critical clerkships and rotations. Our findings suggest that a three-year medical school curriculum can effectively prepare students for residency and produce graduates with comparable medical knowledge and clinical skills, offering potential benefits in terms of financial relief and personal well-being for medical students.

## Introduction

Accelerated Medical School Curricula, specifically three-year programs are not new; however, the most recent resurgence occurred about 10 years ago [1]. Critics of the newer three-year programs cite concern regarding preparedness and impact on wellbeing. An evaluation of the Association of American Medical Colleges (AAMC) Graduation Questionnaire (GQ) revealed that accelerated 3-year pathway program students feel equally prepared for residency compared to their four-year cohorts [2]. Additionally, they also report less debt and no increased rates of burnout. However, evaluations of accelerated pathway programs in terms of performance-based outcomes are still emerging.

The Fully Integrated Readiness for Service Training (FIRST) program at the University of North Carolina School of Medicine is an accelerated and enhanced pathway through medical school, where students complete their undergraduate medical education in 3 years [3]. Completion of the medical school curriculum leads to a directed pathway to an affiliated residency training program, followed by 3 years of service to the state of North Carolina in a rural or underserved setting. In addition to this potential beneficial impact for these communities, accelerated training allows students to reduce the time and financial burdens associated with the long course of medical training. FIRST students may select Family Medicine, General Surgery, Pediatrics, or Psychiatry. Data included in this study represent the first few cohorts of students and therefore are students who selected Family Medicine and Psychiatry.

**Figure 1.**
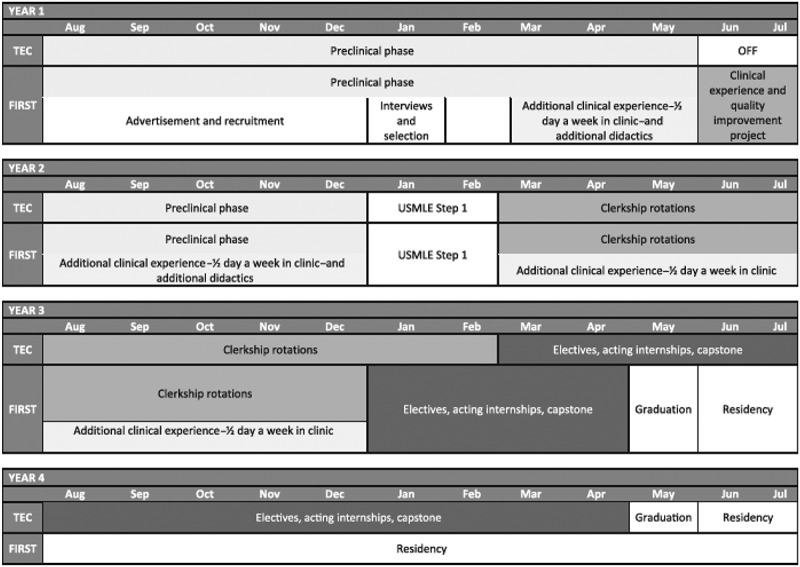
FIRST curriculum overview [3].

Abbreviations: FIRST, Fully Integrated Readiness for Service Training; TEC, Translational Education at Carolina; USMLE, United States Medical Licensing Examination.

During their three-year medical school curriculum, FIRST students are given enhanced clinical experiences, didactics, and mentoring opportunities compared to their traditional four-year curriculum counterparts, Figure 1. Students in the program have dedicated faculty mentors in their specialty of choice, program-specific teaching sessions, and longitudinal clinical experiences starting early in their medical school careers. These additional training experiences help compensate for the condensed curriculum and promote readiness for a successful transition to residency. The goal of the program is to ensure that these FIRST students are equally prepared for residency and showing comparable achievement of medical school competencies as their four-year peers.

To that end, we aim to study and compare measurable outcomes of academic achievement between FIRST students and traditional four-year program students. The objective of this study is to examine the learning outcomes of FIRST students compared to traditional 4-year curriculum medical students. Specifically, we hypothesize that the FIRST students and the traditional curriculum students are equally prepared for residency. The learning outcomes we measured included 1) United States Medical Licensing Exam (USMLE) Step 1, Step 2 Clinical Knowledge (CK), 2) pre-clinical course score, 3) shelf exam scores, and 4) clinical performance scores.

## Materials and methods

This observational study involved retrospective analysis of existing data and was approved by the University of North Carolina Institutional Review Board (IRB # 19–1742).

Details of the curriculum are outlined in the appendix and prior publication [3].

Data from the graduating cohorts of 2018–2023 were included in the analysis. The author, FC, performed the data analysis between May and June 2023. Students who decelerated from the curriculum were excluded from analysis. Students who completed the medical curriculum, but subsequently discontinued the curriculum as a resident were still included for analysis as they had completed the medical curriculum. To assess academic performance comprehensively, we compared the traditional 4-year medical school and accelerated program students on both exam performance and clinical performance measures. The exam performance outcomes included USMLE Step 1 and Step 2 CK, National Medical Board Examination (NBME) shelf exams (e.g., Family Medicine, Surgery, Obstetrics/Gynecology, Pediatrics, Internal Medicine), and Block final exam score percentage (foundation phase courses). Because of the change to passing standard in January 2022, the Step 1 scores of classes who graduated in 2022 or earlier were included [4]. The clinical performance outcomes included evaluation scores from the major blocks at application and individualization phases.

### Analysis

The data were assessed for normality via visual inspection of Q-Q plots and the Shapiro–Wilks test, and use of parametric tests was deemed appropriate. Descriptive statistics were used to summarize responses to demographic items and learning outcomes scores. Chi-square test, Fisher’s Exact test, or two-sample t-test was used in comparing the demographic variables between groups. Analysis of Variance (ANOVA) was used to examine the effect of accelerated pathway program experience relative to traditional 4-year medical school curriculum on the learning outcomes, controlling for class effect. Significance was set at *p* < 0.05. SAS 9.4 (SAS Institute, Cary NC) was used to perform the analysis.

## Results

As summarized in Table 1, except for the mean age upon graduation from the medical school (FIRST: 26.7 ± 2.4, Traditional: 28.3 ± 2.8, *p* = 0.003), there were no statistically significant differences in the major demographic variables (e.g., gender, race, North Carolina resident status, home state, class year) between FIRST students and traditional program students.

**Table 1.** Summary statistics of demographic variables.

	FIRST (*n* = 29)		Traditional (*n* = 1261)		Total (*n* = 1290)		P-value
**Gender, n(%)**							0.588 [1]
Female	17(58.6)		673(53.4)		690(53.5)		
Male	12(41.4)		584(46.3)		596(46.2)		
Not reported*	0(0.0)		4(0.3)		4(0.3)		
**Race, n(%)**							0.103 [2]
American Indian or Alaska Native	0(0.0)		6(0.5)		6(0.5)		
Asian	2(6.9)		199(15.8)		201(15.6)		
Black or African American	1(3.4)		164(13.0)		165(12.8)		
Native Hawaiian or Other Pacific Islander	0(0.0)		2(0.2)		2(0.2)		
White	26(89.7)		725(57.5)		751(58.2)		
Other	0(0.0)		62(4.9)		62(4.8)		
Not reported*	0(0.0)		103(8.2)		103(8.0)		
**NC Resident status, n(%)**							0.242 [2]
Yes	28(96.6)		1105(87.6)		1133(87.8)		
No	1(3.4)		140(11.1)		141(10.9)		
Not reported*	0(0.0)		16(1.3)		16(1.2)		
**NC Home State, n(%)**							
Yes	27(93.1)		1120(88.8)		1147(88.9)		0.762 [2]
No	2(6.9)		137(10.9)		139(10.8)		
Not reported	0(0.0)		4(0.3)		4(0.3)		
**Class of, n(%)**							
2018	2(6.9)		147(11.7)		149(11.6)		0.627 [2]
2019	2(6.9)		131(10.4)		133(10.3)		
2020	2(6.9)		155(12.3)		157(12.2)		
2021	2(6.9)		144(11.4)		146(11.3)		
2022	4(13.8)		146(11.6)		150(11.6)		
2023	7(24.1)		147(11.7)		154(11.9)		
2024	5(17.2)		215(17.0)		220(17.1)		
2025	5(17.2)		176(14.0)		181(14.0)		
**Age upon graduation**							0.003 [3]
N	29		1254		1283		
Mean (SD)	26.69 (2.35)		28.25(2.83)		28.22(2.83)		
Median (Range)	26 (24, 33)		28 (23, 45)		28 (23, 45)		

^a^Chi-Square p-value; ^b^Fisher’s Exact test p-value; ^c^Two-sample t-test p-value; *Not reported cases were excluded from the hypothesis testing.

Abbreviation: *NC – North Carolina*.

Table 2 compares the exam performance of the two groups. There were no significant differences between FIRST and traditional students on any of the NMBE Shelf exams or foundation phase pre-clinical course final exam score percentage. The two groups are also comparable in terms of USMLE Step 1 scores. The only exception is the USLME Step 2 CK score, on which FIRST group averaged slightly lower scores than the traditional group (243.7 ± 17.8 vs 249.1 ± 13.8. *p* = 0.048).

**Table 2.** Comparison between accelerated (FIRST) and traditional students on exam performance.

	FIRST	Traditional	P-value
**PRECLINICAL BLOCK FINAL EXAMINATION**			
**Integumentary Musculoskeletal Final**^**1**^			0.664
N	20	777	
Mean (SD)	87.13 (5.24)	87.41 (6.88)	
**Integumentary Final**^**1**^			0.816
N	4	417	
Mean (SD)	86.51 (8.44)	85.12 (6.92)	
**Musculoskeletal Final**^**1**^			0.866
N	4	419	
Mean (SD)	87.00 (4.16)	86.32 (1.19)	
**Cardiology Final**			0.581
N	24	1194	
Mean (SD)	81.34 (5.62)	82.30 (7.93)	
**Endocrinology Final**			0.268
N	19	1023	
Mean (SD)	87.00 (5.48)	87.59 (7.23)	
**Gastroenterology Final**			0.329
N	24	1194	
Mean (SD)	83.42 (6.50)	84.10 (6.57)	
**Human Behavior and Development Final**			0.759
N	19	1025	
Mean (SD)	88.18 (5.17)	88.30 (6.32)	
**Hematology Final**			0.346
N	24	1194	
Mean (SD)	83.96 (5.81)	84.95 (6.60)	
**Immunology Final**			0.349
N	24	1194	
Mean (SD)	86.33 (4.74)	87.13 (6.05)	
**Multi-organ Synthesis Final**			0.552
N	12	853	
Mean (SD)	85.13 (3.70)	86.21 (6.27)	
**Neurology Final**			0.188
N	18	1024	
Mean (SD)	83.76 (4.95)	85.31 (6.74)	
**Principles of Medicine Final**			0.692
N	24	1194	
Mean (SD)	89.84 (3.29)	89.70 (5.59)	
**Urinary/Renal Final**			0.490
N	19	1026	
Mean (SD)	86.65 (4.55)	87.05 (5.51)	
**Reproduction Final**			0.506
N	12	854	
Mean (SD)	84.90 (3.38)	83.19 (6.76)	
**Respiratory Final**			0.597
N	19	1026	
Mean (SD)	85.63 (4.79)	85.85 (6.65)	
**NBME SHELF EXAMINATION**			
**Family Medicine Shelf**			0.727
N	19	984	
Mean (SD)	79.84 (6.27)	79.96 (6.62)	
**Medicine Shelf**			0.219
N	19	947	
Mean (SD)	75.63 (8.43)	77.44 (7.99)	
**Surgery Shelf**			0.490
N	18	991	
Mean (SD)	74.39 (6.95)	75.21 (7.61)	
**Obstetrics/Gynecology Shelf**			0.329
N	18	984	
Mean (SD)	78.39 (6.81)	79.87 (6.96)	
**Psychiatry Shelf**			
N	18	995	0.551
Mean (SD)	83.78 (4.88)	84.36 (5.72)	
**Pediatrics Shelf**			0.159
N	19	980	
Mean (SD)	77.58 (6.54)	79.57 (7.35)	
**UNITED STATES MEDICAL LICENSING EXAMINATIONS (USMLE)**			
**USMLE Step 1**^**2**^			0.104
N	12	709	
Mean (SD)	222.17 (14.09)	230.09 (18.88)	
**USMLE Step 2 CK**			0.048
N	19	869	
Mean (SD)	243.68 (17.80)	249.07 (13.76)	

P-value based on ANOVA after controlling for Class.

^**1**^Over the years, the Integumentary and Musculoskeletal Courses merged into a single course.

^**2**^Only included Class of 2022 or earlier due to the USLME change to Pass/Fail in January 2022.

Table 3 presents the between-group comparison of the clinical performance during application and individualization phases. There were no significant differences between the two groups in terms of all the included performance metrics.

**Table 3.** Comparison between accelerated (FIRST) and traditional students on clinical performance.

	FIRST	Traditional	P-value
**APPLICATION PHASE**			
**CBLC**			0.072
N	19	892	
Mean (SD)	4.17 (0.38)	4.37 (0.38)	
**HISC Medicine**			0.502
N	18	895	
Mean (SD)	4.37 (0.33)	4.35 (0.37)	
**HISC Surgery**			0.553
N	18	893	
Mean (SD)	4.42 (0.46)	4.34 (0.48)	
**CSP OB/Gyn**			0.550
N	19	889	
Mean (SD)	4.23 (0.43)	4.28 (0.39)	
**CSP Peds**			0.796
N	18	870	
Mean (SD)	4.21 (0.43)	4.21 (0.41)	
**CSP Psych**			0.092
N	18	887	
Mean (SD)	4.46 (0.37)	4.30 (0.39)	
**INDIVIDUALIZATION PHASE**			
**AI**			0.735
N	18	819	
Mean (SD)	4.63 (0.39)	4.58 (0.38)	
**Critical Care**			0.546
N	17	840	
Mean (SD)	4.31 (0.40)	4.39 (0.47)	

Abbreviations:

CBLC – Community Based Longitudinal Care Course – Outpatient, primary care experience.

HISC – Hospital, Interventional, and Surgical Care Course – Inpatient medicine and surgery experience.

CSP – Care of Specific Populations – specialties include Obstetrics/Gynecology, Pediatrics, Psychiatry.

AI – Acting Internship.

## Discussion

Our study sought to evaluate an accelerated medical school program (FIRST) at UNC, to determine whether students completing the pathway were able to maintain similar levels of clinical knowledge and skills as their peers following the traditional four-year medical school curriculum. Students in the FIRST program were not significantly different from peers in preclinical course work scores. While they scored an average of about 5 points lower on Step 2 CK, they had no differences in NBME Shelf exam scores. Most importantly, students received equivalent clinical performance scores in third year clerkships and acting internships and critical care rotations, which are completed by fourth years in the traditional curriculum. The equivalent performance of those in the accelerated pathway mirrors findings from another three-year program [5].

Clinical knowledge was measured in our study by pre-clinical course grades, shelf scores, and Step 2 CK scores. Overall, students in the accelerated pathway did not differ from the traditional cohort in these scores. Because scores evaluating clinical knowledge were equivalent, we suspect that lower scores on Step 2 CK were a result of less dedicated study time for this exam as the FIRST Students took the exam immediately following their core clinical rotations in December of the third year, before transitioning to their final few months of the curriculum in January. Although students in the FIRST Program have the same amount of time to study for the USMLE Step 1 exam, they have about 2–4 weeks less dedicated time to study for their Step 2 exams, compared to the 4-year cohort, due to the condensed curriculum. In addition, students in this curriculum may have less motivation to do extensive study for this exam. Many students in the traditional curriculum rely on Step 2 CK scores to help differentiation in their applications when applying to residency. Given the directed pathway opportunity offered through the FIRST Program, this pressure is likely much less for students in the FIRST program.

Importantly, clinical performance was equivalent when evaluating the students in the FIRST program compared to their traditional program peers. They did not differ significantly from their peers on any third-year rotation evaluation scores. FIRST students scored slightly lower in only the Community Based Longitudinal Clerkship (CBLC) outpatient clerkship, half of which is completed the summer following the first year of medical school. This difference was not statistically significant. Notably, students had equivalent scores in both acting internships and critical care rotations, which are core requirements for fourth year medical students. Based on their evaluations, it appears that FIRST students can perform equivalently to their fourth-year colleagues by the end of the curriculum. This finding mirrors findings from another study evaluating the AAMC GQ data which found students in accelerated pathways felt as prepared as peers for residency [2].

An additional finding of our evaluation is that FIRST students were on average about 1.5 years younger than the traditional group at graduation. Finishing medical school in one less year is financially beneficial to students as they pay one less year of tuition and expenses, and also received income a year sooner. This financial comparison may help students burdened by student loan debt go into lower paying specialties. In the last decades, the dramatic increase of medical education cost has driven post-medical school career choices toward higher compensation specialties in large healthcare systems, discouraging students from applying for primary care specialties and joining practices in rural and underserved communities [6]. This trend has also affected the demographic pattern of the national physician workforce. Several studies showed Black, Native American, Hispanic, and Native Hawaiian/Pacific Islander graduates had a higher rates of debt than their White and Asian counterparts [7]. Prior study shows that graduates of accelerated pathways graduate with less debt and are more likely to go into primary care and care for underserved populations [2]. Admission to medical school and application to residency are a multi-factorial process. However, we think that accelerated pathways may have the potential benefit to promote diversity in the U.S. physician workforce to reflect the national racial and ethnic make-up. Additionally, we hope that graduating at a younger age with a lower debt helps reduce somewhat the burden that many students and trainees feel as they decide when to start families during rigorous medical training [8]. The FIRST students noted they felt lower levels of burnout and stress compared to classmates, given their knowledge of the next steps in their career trajectory and decreased uncertainty.

A limitation of this study is that we are not able to evaluate how students who completed the FIRST program performed during residency. Currently, we do not have enough graduates of the program to meaningfully, and anonymously, compare them to their traditional peers who also stayed at home programs. However, a Canadian study found graduates of their program out-performed traditional curriculum peers from other institutions [9]. Future studies could evaluate intern year clinical evaluations for these students. In addition, future studies might investigate the impact of demographic factors, such as race and ethnicity and contextual factors such as home state, on the accelerated pathway program student’s experience.

## Conclusion

Students in the accelerated pathway graduate with equivalent medical knowledge and skills compared to their peers and are ready for residency training. Students were able to graduate from medical school a year earlier, reducing the financial and time burden of medical education.

## Appendix: Description of FIRST Curriculum

Description of the curriculum and process for implementation is outlined in a publication by Coe, C. et. al^3^. As an accelerated curriculum, the FIRST program incorporates all core aspects of the UNC School of Medicine Translational Education at Carolina (TEC) curriculum while incorporating earlier clinical experiences by minimizing open time in the traditional calendar^3,10^. Students apply for the program in fall and winter of the first year (foundation phase) and are notified of acceptance after interviews with both the core FIRST faculty and participating in statewide residency programs. In spring, FIRST scholars begin incorporating outpatient continuity clinic, in their intended specialties, into their weekly schedules. They also have regular didactics with FIRST faculty on high yield topics, preparing them for this accelerated clinical work. They receive elective credit toward graduation for this clinical and didactic time.

In the summer between the first and second years of medical school, FIRST scholars start the next stage of clinical training with outpatient community medicine (CBLC) at their future residency site for 8 weeks. They then transition back to finish the foundation phase with their classmates in the traditional schedule. They start the application phase in March of their second year at their future residency site. They complete two required 16-week core clinical rotations, ending with the remaining 8 weeks of outpatient medicine.

After completing the core rotations in the application phase, they transition 8 weeks early into the individualization phase with science of medicine, social and health systems, acting internships, and critical care, with early clinical experiences and electives, FIRST scholars can complete the same core curriculum and graduate in May after 3 years.

Throughout the accelerated three-year program, FIRST scholars also receive small group teaching and individualized advising with FIRST faculty as well as residents and faculty at their future residency training sites. These relationships further help enhance preparation for practice in their future communities.
